# COVID-19: An Archetype Innate Immunity Reaction and Modes of Treatment

**DOI:** 10.31138/mjr.31.3.275

**Published:** 2020-09-21

**Authors:** Periklis Vounotrypidis

**Affiliations:** Rheumatology Department, 424 General Military Hospital, Thessaloniki, Greece

**Keywords:** SARS-CoV-2, rheumatology, hyperferritinaemia, immunotherapy, corticosteroids, disease-modifying anti-rheumatic drugs

## Abstract

The magnitude of the SARS-CoV-2 pandemic found health systems unprepared, not allowing for prompt evaluation, collaboration among specialities and treatment of severely ill patients admitted to intensive care units, with many of them having an unfortunate outcome. Current data demonstrate an acute immune dysregulation in severe forms of the disease. The above is concluded by clinical evolution and laboratory findings, indicating a severe inflammatory response of the innate immune system, initiating predominately with the involvement of the respiratory tract epithelial cells, occasionally progressing to thrombotic diathesis and related complications. Besides the clinical manifestations, the immune response expresses an extremely high acute phase reactants repertoire including hyperferritinemia, hyper-fibrinogenaemia, and a storm of cytokines that require an alternative view and collaboration with rheumatologists. Thrombotic diathesis in some cases may not attribute only to a possible disseminated intravascular coagulation, but also to an additional activation of adaptive immunity and the development of the antiphospholipid syndrome. Unifying speciality evaluation and treatment may improve patient outcomes by recognizing early the evolving syndromes, treating properly, in a stratifying manner, with medications that alleviate the inflammatory reaction. Corticosteroids, colchicine, hydroxychloroquine/chloroquine, and possibly potent immunosuppressants are in the armamentarium. Additionally, biologics that interrupt the innate immune dysfunction, such as IL-1, IL-6 and selective JAK inhibitors, are also used. Convalescent plasma therapy and human immunoglobulin may be restricted for those whom the proposed treatments are found inadequate. The above combined with antiretroviral medications may improve the outcome until the development of safe and effective vaccination.

## INTRODUCTION

The reports from the front line of the 2019 Corona Virus Disease (COVID-19), especially from critically ill patients, were initially limited, mainly because of the burden of treating the rapidly increasing numbers of patients. There is an expressed need for a better characterization of COVID-19 infection, especially in severe forms of the disease, in order to guide the decision making.^1^

Much of the therapy and the escalating practices for severe COVID-19 infection are empirical and depended on clinical judgment.^2^ Initially, recommendations were targeting prophylactic measures, screening methods and triage. In severely and critically ill patients, efforts are given to the handling of ventilation, fluid management strategies, and treatment of comorbidities. Careful manipulation is required to avoid medical and unexpected errors such as lung trauma during mechanical ventilation, fluid overload, and related cardiovascular disorders.^2^ Death reasons in COVID-19 severely ill patients have not been broadly or autopsy clarified.^3^ Some may die from the exuberant innate immune reaction, others by their medical comorbidities, organ-specific or multi-organ failures, secondary infections and sepsis, or the lack of means to treatment. This review is an attempt to reveal the degree of innate immunity dysregulation. A stratified immuno-modulating and immunosuppressive treatment approach in patients with COVID-19 is also proposed, based on the acknowledgement of the exaggerated innate-immunity response and the rheumatology practices in similar conditions.

Information based on scientific evidence may occasionally give different views within medical specialities. Additionally, various levels of experience among practitioners of the same speciality may be reflected in patient outcomes. The COVID-19 pandemic is a paradigm where the medical community is called to unify international knowledge and elaborate to improve outcomes and deteriorate losses. Useful information is retrieved from the front-line doctors and the evolving clinical trials.

## CLINICAL PRESENTATION OF SARS-COV-2 INFECTION

The disease can be distinguished into an infectious phase followed in approximately 20% of patients by an overactive immune phase.^4^ The latter is associated with the upregulation of pro-inflammatory cytokines and chemokines and the development of acute respiratory distress syndrome (ARDS)/diffuse alveolar damage. The disease will run mild in the majority of cases. The most common clinical features of COVID-19 infection are summarized in a systematic review and random-effects meta-analyses of the first weeks of the pandemic.^4^ Fever (77%), cough (55.6%), and myalgia (31%) are the most frequent symptoms in adult patients. One-fifth requires hospitalization in intensive care units (ICU), and among them, 38% presented with ARDS (95%CI 13.7–51.8), 13% with acute cardiac injury (95%CI 4.1–21.9), while an acute renal injury or shock presented in 7.9% (95%CI 1.8–14) and 6.2% (95%CI 3.1–9.3), respectively. In the above meta-analysis, lethal outcome had 13.9% (95%CI 6.2–21.5%) of ICU-hospitalized patients.

In severely ill subjects, three types of the disease are identified, classified according to the magnitude of lung injury and the relevant comorbidities.^5^ Type A (22%) comprise those patients with pneumonia, type B (55%) those with different degrees of pneumonia accompanied by severe comorbidities, and type C (23%) the critically ill patients. Appropriate and timely intervention may improve patient outcomes.

Interpretation of current data reveals older age, male sex, smoking, obesity, hypertension, diabetes mellitus, lung disease, cancer and cardiovascular disease as significant risk factors for hospitalization, admission in ICU and/or a lethal outcome.^6–10^ Those who have two or more comorbidities are at even higher mortality risk.^11^ Older age, increased d-dimers and the Sequential Organ Failure Assessment Score (SOFA-score), which is used by internists and emergency medicine doctors are strongly associated with a negative outcome.^10,11^ In a report from China, leading causes of death were: sepsis, respiratory failure, ARDS, heart failure, acute cardiac and renal injury, coagulopathy, and secondary infection.^11^

Interestingly, rheumatic diseases are not included in comorbidities in the majority of studies. The rate of not specified immunodeficiency, as a risk factor, varies in reports from 0.2% to 6%, and is not referred particularly to rheumatic patients.^6,11^ We can assume that there might be a prophylactic potential of anti-rheumatic medications in already treated patients with autoimmune diseases.^12^ The male predominance for severe COVID-19 illness may be explained by an inherent, gender-related overacting innate immunity system in males, in contrast with the well-known, overacting adaptive immunity in females, who are predisposed to diseases with prompt auto-antibody production, such as systemic lupus erythematosus, scleroderma, Hashimoto thyroiditis, and other. Animal studies on SARS-CoV infection have shown an age-dependent innate immune response, with older non-human primates having more intense reactions than younger adults.^13,14^ This is due to a recognised collective loss of immune protection during ageing, which leads to cellular and molecular dysregulation of the innate immune system.^15^ As the pandemic evolves, it is revealed that children are less severely affected, while there is no age or sex preponderance in the paediatric population.^16^ Approximately 2.5% of the laboratory-confirmed cases of SARS-CoV-2 in children may develop severe disease, associated with a cytokine storm similar to that of secondary hemophagocytic lymphohistiocytosis (HLH).^16,17^

The spreading velocity and the ferocity of the infection delayed the attempts of appropriately organized clinical trials for the evaluation of specific treatments. Reasonably, the application of the current knowledge on inflammatory pathways and medications in which rheumatologists are very familiar may contribute to the development of strategic treatment approaches. Case and case series reports are gradually verifying the current rheumatology practice.^18,19^ Antiviral and immunomodulating treatment in SARS-CoV-2 infection should be applied on time to optimize outcomes, similar to the time-depended antiviral treatment for other indications.^20,21^

## A RHEUMATOLOGY APPROACH TO COVID-19

Exaggerated immune responses have always bothered the medical community. It is reflected in the definition of sepsis, which has shifted over time.^22,23^ It mirrors in various names of acute generalized immune responses, where no microbial agent is recognized, such as Severe Acute Respiratory Infection (SARI) and Systemic Inflammatory Response Syndrome (SIRS). It is also reflected in localized-organ oriented, acute systemic responses like the ARDS or Severe Acute Respiratory Syndrome (SARS). The field of innate immunity is among the latest that received the attention of medical society, mainly due to the discovery of new pathways based on interleukin-1 (IL-1) and IL-6 inhibition. Within the rheumatology community, there are views of COVID-19 infection as a disease with rheumatic manifestations or symptoms that mimic rheumatic diseases.^24^ In fact, atypical pneumonia in COVID-19 represents an organ-specific innate immune reaction analogous to those presented in other autoinflammatory diseases, such as the macrophage activating syndrome, the secondary hemophagocytic lymphohistiocytosis or even the Still’s disease. Thus, COVID-19 represents an archetype innate immune dysfunction, an exuberant innate immune response until the development of individuals’ adaptive immunity and antibodies against the SARS-CoV-2.

The innate dysfunction may be recognized by the combination of several elements (**Table 1**): Fever above 38.4° C and acrocytosis, which is interpreted as the extreme number of white blood cells (leucocytosis or most commonly leucopoenia, with prominent lymphopenia), anaemia, or thrombocytopenia.^17^ Additional findings are the extremely high acute phase reactants (ESR and CRP), the hyperferritinemia (greater than x3 the normal value) and the increased fibrinogen and transaminases levels.^17^ The above combination may also be found in sepsis, but elevated procalcitonin values and the negative blood and sample cultures may rule out sepsis. The additional findings of a flawed respiratory membrane performance indicate a severe state of immune dysregulation. This state is expressed with acute and progressive clinical manifestations, such as SARS, SIRS, hemophagocytic syndrome/HLH, which represent different phenotypes of the same procedure.

**Table 1.** Similarities of innate immunity dysfunction in COVID-19 and autoinflammatory syndromes.^58,77,78^

	**COVID-19**	**MAS / HLH**	**AOSD**

**Clinical**			

Fever	> 38.4 °C for > 7 days	>38 °C for > 7 days	>39 °C for > 7 days
Mucocutaneous lesions	Not reported	Mucosal bleeding	Macular, maculopapular, salmon pink
Sore throat	Yes	Not reported	Yes
Arthritis	Not reported	Not reported	Yes
Serositis	Myo/Pericarditis	Myo/Pericarditis	Myo/Pericarditis
Splenomegaly	Not reported	Yes	Yes

**Laboratory**			

ESR	Markedly increased	Markedly increased	Markedly increased
CRP	Markedly increased	Markedly increased	Markedly increased
Acrocytosis	Leucocytosis or more commonly leukopenia – lymphopenia.	Commonly bicytopenias	Leucocytosis
Liver dysfunction	Anaemia, Thrombocytopenia	Anaemia, Thrombocytopenia	Anaemia, Thrombocytopenia
Hyperferritinaemia	Elevated SGOT, SGPT, LDH	Elevated SGOT, SGPT, LDH	Elevated SGOT, SGPT, LDH
Fibrinogen	Yes	Yes	Yes
D-dimmers	Increased	Decreased	Increased
	Increased	Increased	Increased

Abbreviations: COVID-19: Corona Virus Disease 2019; MAS: Macrophage Activation Syndrome;

HLH: Hemophagocytic lymphohistiocytosis; AOSD: Adult Onset Still’s Disease

Furthermore, tissue damage resulting from innate immune responses may trigger pathogenic adaptive immunity reactions, such as the development of antiphospholipid antibodies (APS), increasing the thrombotic diathesis of a patient or entering him in states of emergency due to APS syndrome.^25^ Thrombocytopenic purpura is another abnormal reaction probably of adaptive immunity that follows a SARS-CoV-2 infection.^26^ The combined pulmonary and renal complications in some cases, beyond the septic shock, may also attribute to an emerging vasculitis, similar to small vessel vasculitis that is seen in hypersensitivity vasculitis, Kawasaki disease or possibly in microscopic polyangiitis.^27,28^ An overacting Th2 humoral immunity and the weak clearance of immune complexes by the innate immunity has been proposed as a pathophysiologic event.^29^ Nevertheless, there is a systemic involvement and many organ manifestations in this particular infection.^30^ An additional issue is whether the specific virus has the potential to stimulate the immune system after the supposed recovery and to necessitate the long follow-up of subjects recovered from severe COVID-19 illness, for the early identification of secondary autoimmune diseases. The desirable outcome of patients with COVID-19 is the smooth, gradual development of antibodies against the causative SARSCoV-2 virus. Otherwise, a safe and effective vaccine is the solution for the non-infected population.

Current medical thought on treatment is balancing through hypothetic assumptions and small case-series or case-control studies. The increasing number of fatalities does not allow proper evaluation, which is what concerns internists and the critical care doctors at this particular time, who are in the front lines. Rheumatologists are probably those who may help to unify the knowledge and interpret the immune responses in COVID-19, as well as facilitate treatment procedures, according to the established knowledge and their familiarity of using anti-inflammatory medications. Systemic inflammation that characterizes the severe cases of COVID-19 illness is among the leading causes of death and rheumatologists may help the multidisciplinary treatment of these cases.^31^ A step-up treatment approach should aim at alleviating symptoms and preventing form the transition to the next worse stage of the disease. There are not any particular clinical manifestations or imaging findings early on the disease in SARS-CoV-2 confirmed and non-confirmed cases.^32^ According to published data from laboratory-validated infections, there is a time-dependent worsening of symptoms of patients who will develop progressive disease.^33^ For those who will deteriorate, an average of 7 days is required for admission to the hospital. Furthermore, 8 days are required for the development of dyspnoea, a median of 9.5 days for the development of ARDS, and 10.5 days for the admission to ICU. Escalation must consider clinical and laboratory elements of deterioration. In clinical terms, persistent fever >38, aggravation of cough, the evolvement of dyspnoea, and decrease on SpO2 are considered clinical reasons to proceed to the next therapeutic step. Elevated levels of acute-phase reactants such as erythrocyte sedimentation rate and C-reactive protein, and in particular the increase of those biomarkers indicating an innate immune overreaction, such as ferritin (x3 the normal range) and fibrinogen are justifying further escalation of treatment.^4,34^ This is in accordance with their role as immune regulators who are also present in similar innate immune reactions as the haemophagocytic syndrome, Still’s disease and other autoinflammatory syndromes.^35,36^ Elements of enhanced fibrinolysis, such as d-dimmers and fibrin degradation products (FDP), as well as lymphopenia and increased LDH are considered unfavourable prognostic indicators.^4,33^ Time-dependent interventions are appropriate, as current evidence indicates the progression of the disease in specific time-frames.^33^

## IMMUNOTHERAPY FOR COVID-19

**Figure 1.**
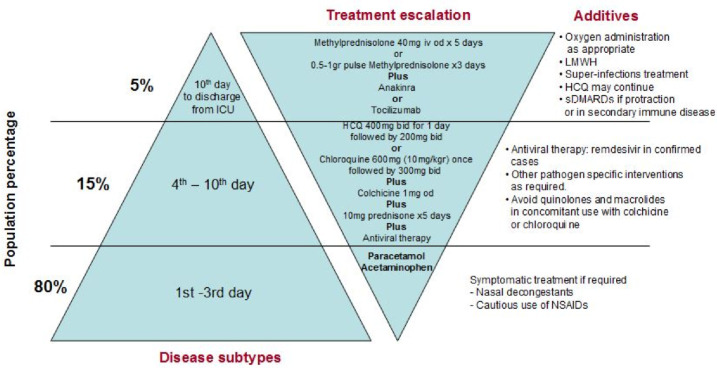
Treatment escalation and graded immunomodulation in SARS-CoV-2 infection. Proposal of the escalating use of immunomodulating treatment, according to the published time-frame of worsening symptoms in COVID-19, in conjunction with the rheumatology practice for systemic autoimmune illnesses. Appropriate collaboration with infectious disease specialists and critical care doctors, in a case by case basis, is advised. Before applying steps of treatment, consider contraindications. Abbreviations: DMARDs: Disease Modifying Anti-rheumatic Drugs; HCQ: Hydroxychloroquine; LMWH: Low Molecular Weight Heparin; NSAIDS: Non-Steroidal Anti-Inflammatory Drugs

Immunotherapy is a mixed, preventive, and curing intervention, depending on the time of application and the mode of treatment. Further to symptomatic therapy, escalating strategies are justified and may apply when symptoms of the disease are prolonged, by using low doses of steroids or otherwise “physiologic” doses (ie, prednisolone ≤10mg/day), on time and in combination with old multipotent drugs with particular anti-inflammatory action such as hydroxychloroquine (HCQ), chloroquine (CQ) and/or colchicine (**Figure 1**).^37–42^ These interactions prevent autoimmunity and decrease tissue damage without immunosuppressing the patient.^43^ There is a delayed therapeutic effect of HCQ and HQ in rheumatic diseases, but in the case of COVID-19 illness, preliminary data suggest an early limitation of radiological progression in COVID-19 subjects treated with HCQ.^44^

Corticosteroid (CS) use has received much criticism for the potential of delaying the virus clearance and the steroid-related side effects.^45^ The World Health Organization (WHO) also, discourages the use of this medication, invoking the lack of data.^2^ Internists are occasionally reluctant to apply the relevant treatment because of the fear of developing secondary infections and the risk of sepsis, but their use is currently revised particularly to those who require critical care.^46,47,48^ Furthermore, low or physiologic doses of steroids may reduce vasopressor requirements and improve the time of shock resolution in patients with sepsis.^49^

Corticosteroids remain the cornerstone treatment for suppressing exaggerating immune responses, and they are the ultimate drugs where every medical speciality resorts to, in difficult inflammatory situations.^17,37,50^ Currently, the developed protocols that include various steroid regimens in treating patients with severe SARS-CoV-2 disease are proving the potential of CS for reducing the number of patients requiring invasive procedures.^51^

Their use undoubtedly mitigates a systemic immune reaction and eliminates tissue damage.^17,37^ Hydrocortisone is commonly applied per protocol in intensive care units, but formulations that combine glucocorticoid and mineralocorticoid action, such as methylprednisolone, may improve clinical and laboratory parameters and eliminate the need for intense oxygen support or mechanical ventilation in COVID-19 patients.^52^ In this particular infection, their benefit was unequivocal and was proposed as adjuvant therapy (1–2 mg/kg body weight) in the 5^th^ revision on the treatment of the severe 2019-nCoV disease, by China’s National Health Organization.^37,53^

Similar to rheumatology practices, increased steroid regimens (40–60 mg Methylprednisolone daily) must be taken into account in severe cases of COVID-19, by the time that x-ray aggravation is conceivable and before the admission to ICU. Alternatively, pulse steroids (0.5–1 gr methylprednisolone/day for 3 consecutive days) may be used when further deterioration occurs, in a similar manner to other hyperferritinaemic or exaggerated autoimmune diseases (**Figure 1**).^54,55^ Gradual down-regulation and discontinuation of steroid treatment can be considered by the time of clinical improvement in parallel with normalization of acute-phase reactants.

Cytokine-directed therapies may be required as an adjuvant treatment, in case of persistent symptoms or worsening of the clinical, laboratory and radiological findings. Interleukin-1 plays a critical role in developing hypoxaemia and increased lung permeability; thus, its inhibition is justified in the case of severe SARS-CoV-2 infection.^56,57^ Monteagudo et al. used the IL-1 receptor antagonist anakinra in up to 2400mg daily, on a continuous IV infusion in patients with MAS, anticipating that large amounts of IL-1 inhibition may be beneficial to unresponsive patients in the case of severe COVID-19 disease.^58^

Interleukin-6 (IL-6) is a pivotal cytokine in acute phase responses, and its inhibition may alleviate acute innate-immunity reactions and the related cytokine storm.^59,60^ IL-6 levels are correlated to the disease severity and the viral load in patients with COVID-19.^61^ They are also predicting the need for mechanical ventilation when combined with elevated levels of CRP.^62^ As it has been shown in other hyperferritinaemic inflammatory syndromes, the use of tocilizumab as a complementary therapy may be beneficial in severe cases of COVID-19.^63,64^

Some of the newest medications, selective Janus-kinases (JAK), have shown a potential for combination with antiviral agents due to their ability to reduce viral infection in vitro and minimizing the host response, by eliminating over-secretion of interferon-γ.^65,66^ Biologic treatments have the advantage of quick and targeted action, are less toxic than chemotherapeutic therapies, and the potency for combining treatments with minor regimens of steroids and synthetic disease-modifying drugs. Ongoing trials may prove the rate of effectiveness in the case of severe SARS-CoV-2 infection.

For protracted symptoms, a revision of the treatment may be required among with the investigation of the development of a secondary autoimmune disease (ie, APS or vasculitis). At that time, discontinuation of the biologic DMARD and the addition of a synthetic DMARD, according to the current rheumatology practices, such as cyclosporine, mycophenolate mofetil, azathioprine or a pulse of cyclophosphamide (0.5–1gr), may be considered appropriate.^67,68,69^ This is justified when treating hyper inflammation, by using existing approved therapies to address the immediate need to eliminate the rising mortality, but certainly needs validation from clinical trials.^17^ Prospective trials may reveal the best treatment options, but at this time of emergency, the application of essential medicine is required according to the current knowledge and the practices in which rheumatologists are most familiar.

Convalescent plasma therapy constitutes a passive immunization, an intravenous infusion of plasma received from recovered patients with COVID19 infection.^70,71^ Studies have shown that 200ml of plasma received from recovered donors improves clinical and laboratory parameters when injected in patients with severe disease.^70,71^ It is probable that newly formed antibodies against SARS-CoV-2, which means an adaptive immunity intervention, minimize the viral load, allowing time for a patient to recover. A recent review on the use of convalescent plasma and hyperimmune immunoglobulin identified, so far, low-certainty evidence on the effectiveness and safety of these methods.^72^

Another treatment approach, based on the rational similarities between COVID-19 and high altitude pulmonary oedema (HAPE) proposing the use of acetazolamide, nifedipine and phosphodiesterase inhibitors.^73^ Despite the criticism that has been received,^74^ this treatment resembles that of pulmonary hypertension, which may also be present in systemic inflammatory diseases and over-coagulating states and worth validation in suspected cases.

Finally, there is a need to reveal any possible prophylactic potential of the antirheumatic medications against COVID-19 to already treated patients with various rheumatic diseases. The Rheumatology community and validated registries could offer valuable information.

## CONCLUSIONS

There is time to conceptualise the treatment of COVID-19 on a new basis, adding the experience of rheumatologists in managing the escalating symptoms of the disease. Besides the urgent need for effective drugs, there is an additional requirement of improving performances by using old drugs, especially proper handling of corticosteroid treatment and the already tested therapies, which must not act as competitors to newest, targeted and expensive treatments. As it has been shown in other rheumatic diseases, treatment strategies are important using available drugs.^75^ Unchartered inflammatory processes in COVID-19 infection should not take us away from the established knowledge of immune reactions, and the disease-forming processes of innate immunity. What was a once- or twice-a-year case for a rheumatology department of a medium-size hospital, is now a massive phenomenon that overwhelms intensive care units and paralyzes health care systems. The stratification of medical approaches is urgent under the current knowledge. It took much time for rheumatologists to make primary care physicians and orthopaedic surgeons aware of the necessity of early recognition of arthritis and the need for early reference and intervention, in order to improve outcomes. Similarly, there is an urgent need for involvement of rheumatologists in decision-making for treatments, especially in severely ill patients with SARS-CoV-2 infection. This particular infection will help us in the future to understand better the mechanisms of “aseptic” sepsis.

The use of immunomodulating and immunosuppressive strategies on timely interventions could minimize the percentage of critically in patients and benefit outcomes of the systemic illness, giving time and allowing adaptive immunity to respond appropriately. The affirmative and protective action of small doses of steroids and the concomitant use of cheap multipotent anti-rheumatic drugs HCQ and colchicine are unequivocally beneficial to some patients. Aggressive approaches with pulse steroid treatment and selective cytokine inhibition with biologics would further benefit the severely ill and the initially non-responders. More potent disease-modifying anti-rheumatic drugs could be used in subjects with protracting symptoms and secondary development of adaptive immunity-related nosologies, such as idiopathic thrombopenic purpura, antiphospholipid syndrome, Kawasaki disease, or ANCA-associated vasculitis. Human immunoglobulin treatment, due to its limited availability, must be reserved for particular cases and young patients when other options have failed. The proposed escalating approach on gradually aggressive cases of COVID-19, is on the shadow of the ongoing clinical trials, waiting for their results on treatment effectiveness as well as the determination of predisposing genetic factors to particular immune reactions.^76^

## ABBREVIATIONS

ARDS: Acute Respiratory Distress Syndrome
ANCA: Anti-Neutrophil Cytoplasmic Antibody
COVID-19: Coronavirus Disease 2019
CRP: C-Reactive Protein
CS: Corticosteroids
HAPE: High Altitude Pulmonary Oedema
HCQ: Hydroxychloroquine
HLH: Hemophagocytic Lymphohistiocytosis
HQ: Chloroquine
ICU: Intensive Care Unit
JAK: Janus Kinase
NSAIDS: Non-Steroidal Anti-inflammatory Drugs
SARI: Severe Acute Respiratory Infection
SARS-CoV-2: Severe Acute Respiratory Syndrome-Coronavirus-2
SIRS: Systemic Inflammatory Response Syndrome
